# Functionalized rGO Interlayers Improve the Fill Factor and Current Density in PbS QDs-Based Solar Cells

**DOI:** 10.3390/ma12244221

**Published:** 2019-12-16

**Authors:** Anton A. Babaev, Peter S. Parfenov, Dmitry A. Onishchuk, Aliaksei Dubavik, Sergei A. Cherevkov, Andrei V. Rybin, Mikhail A. Baranov, Alexander V. Baranov, Aleksandr P. Litvin, Anatoly V. Fedorov

**Affiliations:** Center of Information optical technology, ITMO University, 197101 St. Petersburg, Russia; psparfenov@itmo.ru (P.S.P.); onishchuk.d@itmo.ru (D.A.O.); adubavik@itmo.ru (A.D.); s.cherevkov@itmo.ru (S.A.C.); andrei.rybin@gmail.com (A.V.R.); mbaranov@mail.ru (M.A.B.); a_v_baranov@yahoo.com (A.V.B.); litvin@itmo.ru (A.P.L.); a_v_fedorov@mail.ifmo.ru (A.V.F.)

**Keywords:** solar cells, reduced graphene oxide, quantum dots, impedance spectroscopy

## Abstract

Graphene-quantum dot nanocomposites attract significant attention for novel optoelectronic devices, such as ultrafast photodetectors and third-generation solar cells. Combining the remarkable optical properties of quantum dots (QDs) with the exceptional electrical properties of graphene derivatives opens a vast perspective for further growth in solar cell efficiency. Here, we applied (3-mercaptopropyl) trimethoxysilane functionalized reduced graphene oxide (f-rGO) to improve the QDs-based solar cell active layer. The different strategies of f-rGO embedding are explored. When f-rGO interlayers are inserted between PbS QD layers, the solar cells demonstrate a higher current density and a better fill factor. A combined study of the morphological and electrical parameters of the solar cells shows that the improved efficiency is associated with better layer homogeneity, lower trap-state densities, higher charge carrier concentrations, and the blocking of the minor charge carriers.

## 1. Introduction

Many research groups are attempting to improve the efficiency and stability of lead sulfide colloidal quantum dots (QDs) based solar cells [1,2,3,4]. In addition to being tunable throughout the NIR region bandgap, low-cost solution processing and the capacity for multiple exciton generation make lead sulfide QDs a highly suitable material for solar cells. Currently, 12% power conversion efficiency (PCE) is the record for heterojunction PbS QDs-based solar cells [5]. However, it is still beyond the theoretical limits of this material [6].

Defects in the active layer are among the main sources of charge carrier losses in solar cells. In solar cells based on QDs, the process of ligand replacement causes a rise in the defects in the active layer. Long ligands are replaced by short ones, which leads to the rearrangement of QDs and, as a result, to the formation of cracks and pinholes [7]. Nonetheless, the interdot spacing and layer homogeneity are crucial for layer conductivity [8]. In a number of works, graphene and its derivatives were applied to improve the parameters of solar cells’ active layers [9,10,11,12,13], including morphology.

Graphene and its derivatives possess an excellent charge mobility and adjustable properties, which make them a promising material for photovoltaic applications [14]. Recently, Beatriz Martín-García et al., presented a noticeable technique [15] of reduced graphene oxide (rGO) functionalization by (3-Mercaptopropyl) trimethoxysilane (MPTS) for rGO-PbS QD linking that has vast perspectives for device fabrication. Later, we showed that a preliminary colloidal-phase ligand exchange allows a higher efficiency of charge transfer between MPTS-bonded QDs and rGO to be achieved [16]. Additionally, it was shown that a thin layer of MPTS-bonded rGO-PbS deposited on top of a hole-transporting layer improves the stability of a solar cell [17].

Here, we show that MPTS-functionalized rGO (f-rGO) can be inserted into the active layer of a PbS QDs-based solar cell. The interlayers of f-rGO drastically boost the performance of a heterojunction PbS QDs-based solar cell due to increased current density and improved fill factor (FF).

## 2. Materials and Methods

### 2.1. Materials

Precursors PbO (99.999%), hexamethyldisilathiane (TMS), zinc acetate dehydrate (99.99% Aldrich, Saint Louis, MO, USA), MAI (methylammonium iodide), EDT 1,2-ethanedithiol, TBAI (tetrabutylammonium iodide), and DMF (dimethylformamide anhydrous) were purchased from Sigma-Aldrich (Saint Louis, MO, USA); acetonitrile from AppliChem (Darmstadt, Germany); octadecene (ODE) was obtained from Acros (Geel, Belgium); oleic acid (OlAc) from Fisher Chemicals (Waltham, MA, USA); EtOH (ethanol), MeOH (methanol), and chloroform were purchased from Vekton (St. Petersburg, Russia). Reduced graphene oxide (rGO) stabilized with poly (sodium 4-styrenesulfonate) was purchased from Sigma-Aldrich as an aqueous solution and used as received. The 95% (3-Mercaptopropyl) trimethoxysilane (MPTS) was purchased from Sigma-Aldrich.

PbS QDs were synthesized using the hot-injection method described elsewhere [18,19]. Briefly, 0.088 g of PbO was dissolved with 0.26 mL OlAc in 3.8 mL ODE at 90 °C under vacuum as a lead precursor. The sulfur precursor was prepared by dissolving 70 μL TMS in 2 mL ODE. The sulfur precursor was swiftly injected in the lead precursor at 85 °C, heated for 2 min, and cooled using a water-bath. The resulting QDs with a diameter of 3.5 nm were obtained by washing them with acetone and centrifugation at 6000 RPM. The PL and absorption spectra of QDs are shown in Supplementary Materials Figure S1.

For the MAI phase ligand-exchange process, 1.5 mL of a 0.04 M MAI in 2:1 toluene-DMF solution was added at the rate of (3 µL·s^−1^) to 4 mL of the PbS QD solution with a concentration of 50 mg/mL, under gentle stirring. The resulting solution was kept under an inert atmosphere for 12 h to complete the exchange. Following this, MeOH was used to precipitate the solution, and finally, the solution was redispersed in octane [20]. The solution in octane was ultrasonicated for 15 min and filtered through a 0.22 µm syringe hydrophobic filter prior to use.

The method applied by Beatriz Martin-Garcia et al. [15] was used for rGO functionalization. Briefly, 10 mL of 0.5 mg/mL rGO dispersion in EtOH was sonicated for 30 min and refluxed at 60 °C for 15 h with 1.25 mL of MPTS. The resulting mixture was washed with EtOH by centrifugation and finally dispersed in EtOH by sonication for 30 min. The solution was additionally sonicated for at least 15 min just before use.

ZnO nanoparticles were synthesized according to the literature with some modifications [21]. Thirteen millimoles of zinc acetate dehydrate was dissolved in 125 mL of methanol at 60 °C. Twenty-six millimoles of potassium hydroxide was dissolved in 65 mL of methanol. The potassium hydroxide solution was slowly added to the zinc acetate solution and the solution was left stirring at 65 °C for 3 h. ZnO nanocrystals were extracted by centrifugation and then washed twice with methanol followed by centrifugation. Finally, 10 mL of chloroform and 10 mL of methanol were added to the precipitates and the solution was filtered with a 0.45 mm filter.

### 2.2. Device Fabrication

The devices were fabricated following the layer-by-layer spin-coating method according to [22]. Fifty nanometers-thick ZnO layers were deposited on the clean pre-patterned ITO-coated glasses at 3000 rpm and annealed at 150 °C for 30 min. After cooling to room temperature, 6 layers of MAI-treated QDs from 50 mg/mL octane solution were deposited at 2500 rpm on the ZnO film with a 2-step ligand-exchange procedure for each layer, using 15 mg/mL TBAI in methanol (MeOH) solution for exchange and acetonitrile (ACN) and hexane (HEX) for washing. Furthermore, 2 layers of OA-capped PbS QDs were deposited from a HEX solution at 2500 rpm on the TBAI layers, with ligand exchange using 0.1% EDT in ACN as an exchange solution and ACN and HEX for washing. Finally, a 100 nm Au electrode was deposited by magnetron plasma sputtering.

### 2.3. Measurements

The atomic force microscopy (AFM) measurements were provided using a Solver PRO-M microscope (NT-MST, Moscow, Russia) in the semi-contact mode. The scanning electron microscopy (SEM) measurements were performed using a Merlin Zeiss electron microscope (Carl Zeiss, Oberkochen, Germany) in high vacuum mode at 10 kV accelerating voltages.

The light and dark J–V characteristics were measured by an Ossila Solar Cell I-V Test System. A solar simulator was used based on an OSRAM XBO-150W/1 xenon lamp (OSRAM, Munich, Germany). The light intensity adjusted was 100 mW/cm^2^. Capacitive and frequency characteristics were measured by a Keysight E4980A LCR Meter (Keysight Technologies, Santa Rosa, CA, USA).

## 3. Results and Discussion

The conventional ITO-ZnO-PbS(TBAI)-PbS(EDT)-Au solar cell architecture, with MAI passivation QDs for the TBAI layer, was employed for our study. The preliminary colloidal-phase MAI of PbS QDs surface has been recently used to enhance the performance of PbS-based solar cells [20]. Additionally, we have recently shown that such a ligand exchange provides better charge transfer in rGO-PbS QD composites [16].

**Figure 1:**
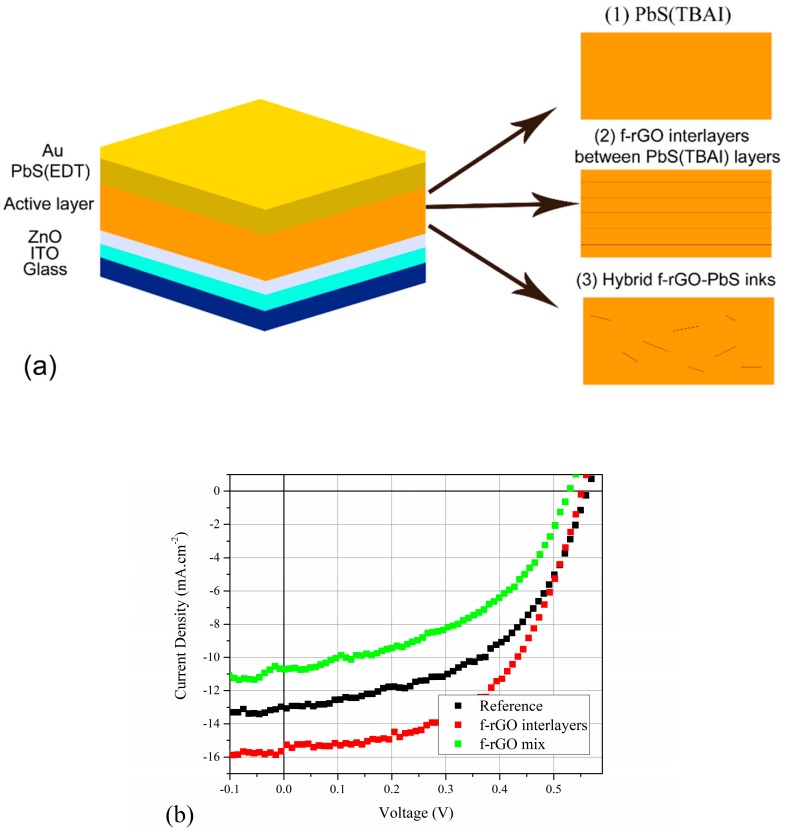
(**a**) Schematic illustration of the reference (Device 1), interlayers (Device 2), and hybrid structures (Device 3); (**b**) J–V curves of the devices.

Devices with the three types of the active layer were explored, as shown in Figure 1a. For the reference device (Device 1), only MAI-treated QDs were employed for the active layer fabrication. For the f-rGO interlayers device (Device 2), the same conditions for Device 1 were used, but two drops of the 0.05 mg/mL f-rGO in EtOH solution were additionally spin-coated on the interfaces between the TBAI-treated PbS layers at 3000 rpm. For the device with an f-rGO-PbS hybrid in the active layer (Device 3), 0.5 mL of 50 mg/mL MAI-treated PbS colloidal solution in octane was mixed with 0.05 mL of 0.5 mg/mL f-rGO in EtOH solution by stirring for 30 min at 2000 rpm. These hybrid inks were employed instead of QD inks for the fabrication of six TBAI layers in Device 3 with the same deposition and ligand-exchange conditions as for Device 1. The PbS(EDT) hole transport layer remained the same for all the devices. The light parameters of the devices are represented in Table 1.

Figure 1b presents the J–V curves obtained for the three types of devices. As seen, the devices with f-rGO interlayers possess better current, while the f-rGO mixture device suffers current quenching, and both devices exhibit slight V_OC_ reduction. Moreover, Device 2 has a significant FF improvement. The FF, short circuit current density (J_SC_) and open circuit voltage (V_OC_) values are given in Table S1. The AFM, capacitance–voltage (C–V), and capacitance–frequency (C–F) measurements were performed to reveal the nature of the changes.

The AFM measurements were performed to examine the influence of the rGO on the film quality. The following samples were compared:

(1) 2-layer sample of TBAI-treated QDs;
(2) 2-layer sample of TBAI-treated QDs with rGO interlayer;
(3) 2-layer sample of TBAI-treated QDs with f-rGO interlayer;
(4) 2-layer sample of TBAI-treated layers from f-rGO-PbS hybrid inks.

**Figure 2:**
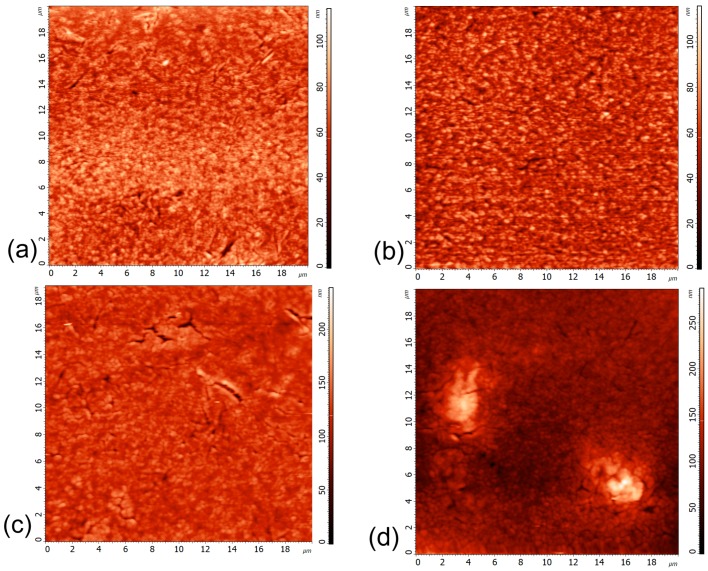
Typical atomic force microscopy (AFM) images (**a**) tetrabutylammonium iodide (TBAI) treated quantum dots (QDs); (**b**) TBAI-treated QDs with rGO interlayer; (**c**) TBAI-treated QDs with f-rGO interlayer, and (**d**) TBAI-treated layers from f-rGO–PbS hybrid inks.

The samples obtained were measured on 20 × 20 µm of 10 randomly selected areas on each sample, and average roughness (R_a_) data were calculated by NT-MDT NOVA image analysis software and averaged on the measured points. The examples of AFM images are shown in Figure 2, and the average roughness data are given in Table S2. Additionally, the listed structures were studied using SEM (Figure S2). The lower average roughness indicates a lower amount of cracks and pinholes. From the data, it follows that the f-rGO interlayers help minimize the quantity of the defects in the planar PbS layer-by-layer film, while using f-rGO–PbS hybrid inks results in huge aggregates in the film. In contrast, the non-functionalized rGO layer corrupts the film quality, which confirms that the MPTS linker attached to rGO plays a key role in the improvement of the films’ morphology.

The R_a_ correlates with the FF and J_SC_ of the devices and is totally independent from V_OC_ change. V_OC_ is sensitive to the charge carriers’ recombination [23,24], which could be enhanced by the rGO incorporation, while the better the film quality the better the QD layer resistance, thus increasing the current and FF.

The dark J–V characteristics were measured for Devices 1–3, as shown in Figure S3. The resistances and ideality factors of the devices were obtained from these curves. The ideality factors are in the range of 3.5 ± 0.3, which is typical for the devices of this type [25,26,27]. The ideality factors and other device parameters are given in Table 2.

**Figure 3:**
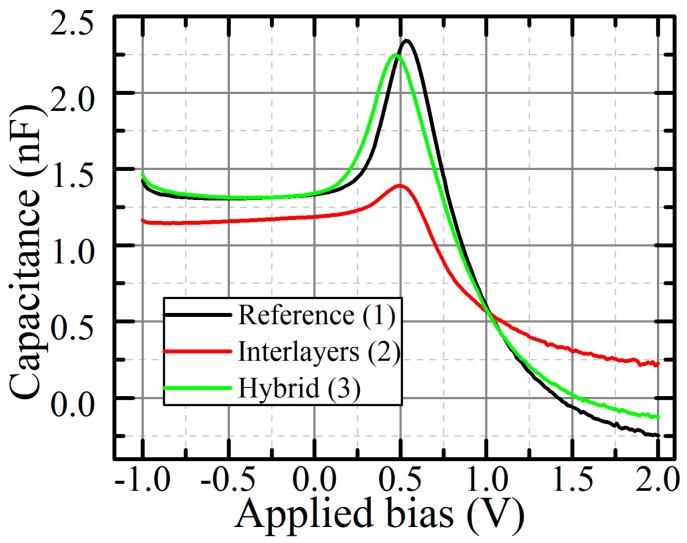
Capacitance of the devices at different bias conditions.

Next, the dark C–F characteristics were obtained, as shown in Figure S4. The contribution of deep traps to the device capacitance is minimal in flat areas [28]; therefore, we chose a frequency of 10 kHz—a relatively flat frequency—for all the devices to measure C–V. The C–V characteristics obtained are shown in Figure 3.

From the minimum capacitance in Figure 3, which is observed at a negative bias, the dielectric constant can be determined from the equation for the capacitance of a flat capacitor, $C_{dep}=\frac{{\varepsilon \varepsilon }_{0}S}{W_{dep}}$ [29], where C_dep_ is the measured capacitance, ε is the relative dielectric constant, *S* is the pixel area, W_dep_ is the depletion region width of the device that equals the geometric thickness when the device is fully depleted, and ε_0_ is the vacuum permittivity. The reverse bias at which the capacitance reaches the minimum was −0.5 V, −0.9 V, and −0.4 V for 1, 2, and 3 devices, respectively. The calculated dielectric constants of f-rGO devices are slightly lower than the value obtained for Device 1, whereas the addition of material with a higher dielectric constant should increase the total dielectric constant. With a large forward bias, the capacitance of Device 2 stays positive, while the capacitance of the remaining devices becomes negative. This indicates the transition of the capacitance into the inductance. The inductance effect is present in diodes, due to the modulation of the layers’ conductivity, when the density of minority charge carriers exceeds a certain part of the main density of charge carriers [30]. Thus, we believe that the difference at a high forward bias may occur due to the blocking of minor charge carriers by f-rGO layers.

**Figure 4:**
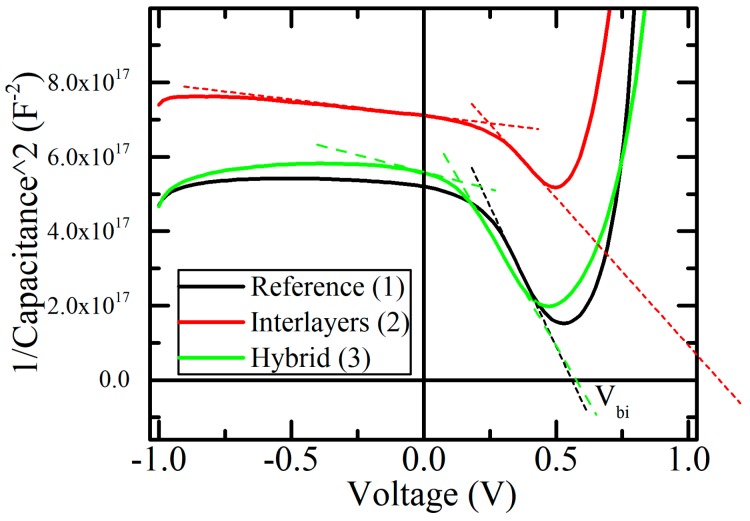
Mott-Schottky plot.

Figure 4 shows the Mott-Schottky plot, which is described by Equation (1):

(1) $$\frac{1}{C^{2}}=\frac{2}{{q\varepsilon N}_{D}}\left(V_{bi}+V_{r}\right)$$

where ε is the relative dielectric constant; V_bi_ is the built-in potential; V_r_ is the applied voltage; N_D_ is assumed to be the concentration of charge carriers. Generally, the N_D_ value in Equation (1) is the density of uncompensated donors or acceptors [31], but in our case, charge carrier transport in PbS-TBAI is dominated by electron transport since PbS-TBAI shows n-type behavior [32,33,34]; therefore, we assume N_D_ as the concentration of free charge carriers (electrons).

When C^−2^ tends to 0, V_bi_ = V_r_; thus, extrapolating the curve C^−2^ to zero, we can obtain the values of V_bi_. As one can see from Figure 4, the V_bi_ obtained this way for Device 2 is much larger than for the other devices. However, this is unlikely because the V_bi_ value is usually determined by the material of the electrodes and the interfaces on the electrodes, which remain the same for all the devices. Additionally, a real change in V_bi_ should induce a change in the position of the capacitance peak [34]. Apparently, as seen in Figure 4, the position of the peak is approximately the same for all the devices. For this reason, V_bi_ was taken as the constant and was determined directly from the Mott-Schottky plot of Device 1. The error in the determination of V_bi_ is associated with the rise in the curve of Device 2 on the Mott-Schottky graph, which can be explained by the appearance of additional capacitance, which is connected to the main junction in series.

If the additional capacitance is in the active layer, the effect of series capacitance should maximally emerge at a forward bias and be minimal at a reverse bias, since the energy levels at which the charge accumulates are also depleted in a reverse bias and filled in a forward bias. This is confirmed by Figure 4, where the minimum difference is observed in the fully depleted mode, and the maximum difference is observed when the depletion zone has a minimum width. Additional capacitance can come from different sources, for example, an oxide layer [35] or deep traps [36]. We believe that it arises due to the introduction of f-rGO layers. Thus, the appearance of additional capacitance leads to the underestimation of the total capacitance measured for Device 2, which explains the lower calculated value of the dielectric constants.

The C^−2^ curve of Device 2 in Figure 4 demonstrates a flatter slope at the reverse bias. Such a slope is usually associated with the existence of deep traps, which inject charge carriers even with the depletion region reaching the electrodes [35]. Thus, the sequential f-rGO deposition leads to the appearance of deep traps, which act as an additional capacitance. Another effect of the f-rGO introduction into the active layer of devices is an increase in the concentration of charge carriers, which can be determined from the angle of inclination of the curve C^−2^ from the graph in Figure 4. The values calculated are shown in Table 2. The increase in the concentration of charge carriers correlates with an increase in the efficiency of the devices observed from the light J–V characteristics.

The density of states inside the band gap can be calculated from the С–F dependence shown in Figure S4 as follows:

(2) $$g\left(E\right)=-\frac{V_{bi}}{{qwk}_{B}T}\times \frac{dC\left(\omega \right)}{dLn\left(\omega \right)}$$

where C is the capacitance; ω is the angular frequency; w is the width of the depleted zone; k_B_ is the Boltzmann constant; T is the temperature in Kelvins; E is the energy associated with the applied frequency by the following Equation (3):

(3) $$E_{\omega }=\frac{k_{B}T}{q}Ln\left(\frac{\omega_{0}}{\omega }\right)$$

where ω_0_ = 2π*v*_0_, and *v*_0_ is a constant, which for the devices can be considered equal to *ν*_0_ = 2.88 × 10^12^ s^−1^ [37]; E_ω_ is the energy level depending on the type of material (E_V_ for p-type and E_C_ for n-type).

**Figure 5:**
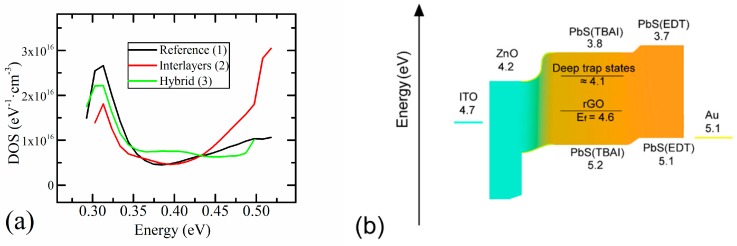
(**a**) Density of the states and profile of the devices (g(E)), and (**b**) energy levels and structure of the devices.

The plot of g(E) is shown in Figure 5a. The pronounced peak in the plot of g(E) in the region of 0.30–0.35 eV corresponds with the deep trap states of PbS QDs [37]. These states corrupt the efficiency of the device and their contribution increases with the degradation of QDs caused by atmospheric air [34]. As seen from Figure 5a, the density of states in the region of 0.30–0.35 eV is much lower for both devices with f-rGO. It is known that rGO [17] and other allotropic forms of carbon [38] increase the stability of photovoltaic devices with employed architecture due to the protection from atmospheric moisture. This correlates with our results because the devices were fabricated and measured in ambient conditions.

For Device 2, a significant increase in the density of trap states in the energy region above 0.45 eV is observed. This is in agreement with the results obtained from C–V measurements, which indicate the appearance of additional deep traps in Sample 2. It should be noted that the influence of the middle bandgap states on the device efficiency is lower than the influence of trap states between 0.30 and 0.35 eV, since the rate of trap state emission exponentially depends on their depth [28]. Nevertheless, such states affect the V_OC_ [23,39] via trap-assisted recombination, which correlates with the results obtained for our devices from the light J–V characteristics and slight n growth.

## 4. Conclusions

We have demonstrated different approaches to embed the functionalized rGO in the active layer of PbS QDs-based solar cells. Employed as interlayers between TBAI-treated layers, functionalized rGO improves the fill factor and current density in PbS QDs-based solar cells. By using AFM, C–V, and C–F analysis, we have shown that such an increase in device performance is induced by the optimization of the active layer quality via arranging the QDs’ layer growth, having a higher concentration of charge carriers, and by blocking the minor charge carriers. Additionally, analyzing the density of states revealed the reduction of trap states between 0.3 and 0.35 eV associated with QD degradation. The method demonstrated is universal and can be applied elsewhere to fabricate QD multilayer solids.

## Figures and Tables

**Table 1:** The fill factor (FF), J_SC_, and V_OC_ values of the reference, functionalized reduced graphene oxide (f-rGO) interlayers, and f-rGO mix devices.

Device	FF (%)	J_SC_ (mA⋅cm^−2^)	V_OC_ (V)	PCE (%)
Reference (1)	48.4 ± 3.5	13.5 ± 1.2	0.57 ± 0.005	3.5 ± 0.4
Interlayers (2)	54.2 ± 0.3	14.2 ± 1.3	0.55 ± 0.005	4.2 ± 0.35
Hybrid (3)	42.6 ± 6	10.8 ± 0.2	0.54 ± 0.018	2.5 ± 0.4

**Table 2:** The calculated characteristics of the devices.

Sample	Relative Permittivity ε	N_D_ (cm^−3^)	n	V_bi_ (V)	R_S_ Dark|Light (Ohm × cm^2^)	R_SH_ Dark|Light (kOhm × cm^2^)
Referance (1)	19.8	5.3 × 10^15^	3.3	0.57	2.8|11.0	0.86|0.12
Interlayers (2)	16.4	10.4 × 10^15^	3.8	0.57	4.3|17.5	0.82|0.17
Hybrid (3)	18.9	6.7 × 10^15^	3.5	0.57	2.4|13.0	0.75|0.28

## Supplementary Materials

The following are available online at https://www.mdpi.com/1996-1944/12/24/4221/s1, Figure S1: the absorption and PL spectra of PbS QDs., Figure S2: The SEM images of TBAI treated QDs, TBAI treated QDs with rGO interlayer, TBAI treated QDs with f-rGO interlayer and TBAI treated layers from f-rGO–PbS hybrid inks, Figure S3: the dark I-V curves of the devices, Figure S4: capacitance of the devices at different frequencies, Table S1: The measured average roughness of the samples with and without rGO interlayer.
