# Supplementary material for: Functionalized rGO Interlayers Improve the Fill Factor and Current Density in PbS QDs-Based Solar Cells
Source: Materials (Basel). 2019 Dec 16;12(24):4221. doi: 10.3390/ma12244221 (PMC6947317; doi:10.3390/ma12244221)
Supplement: Supplementary file 1 [file materials-12-04221-s001.pdf]

materials

Article

Functionalized rGO Interlayers Improve the Fill Factor and Current Density in PbS QDs-Based Solar Cells

Anton A. Babaev *, Peter S. Parfenov, Dmitry A. Onishchuk, Aliaksei Dubavik, Sergei A. Cherevkov, Andrei V. Rybin, Mikhail A. Baranov, Alexander V. Baranov, Aleksandr P. Litvin, and Anatoly V. Fedorov

Center of Information optical technology, ITMO University, 197101 St. Petersburg, Russia; od@mail.ifmo.ru; psparfenov@itmo.ru (P.S.P.); onishchuk.d@itmo.ru (D.A.O.); adubavik@itmo.ru (A.D.); s.cherevkov@itmo.ru (S.A.C.); mbaranov@mail.ru (M.A.B.); andrei.rybin@gmail.com (A.V.R.); a_v_baranov@yahoo.com (A.V.B.); litvin@itmo.ru (A.P.L.); a_v_fedorov@mail.ifmo.ru (A.V.F.)

* Correspondence: a.a.babaev@ifmo.ru or a.a.babaev1@gmail.com

The QDs PL and absorption spectra are imaged in Figure S1. The QDs solution were measured employing Shimadzu UV-3600 spectrophotometer and self-constructed experimental setup for NIR-PL measurements described in [1]. The size was calculated from the absorption spectra employing the relation from [2].

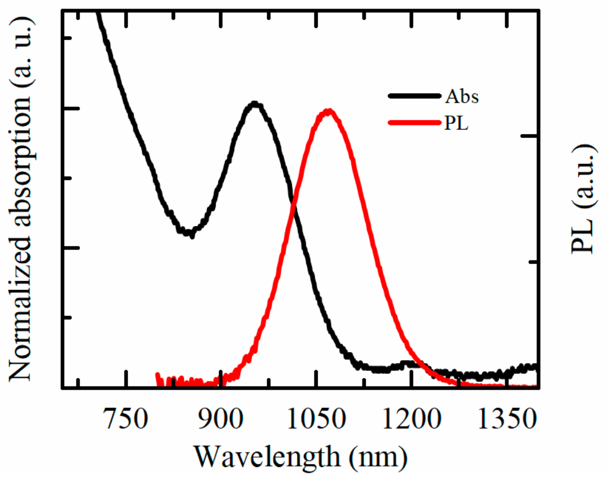

Figure S1. The absorption (black) and PL (red) spectra of PbS QDs.

The analysis of the 2-layered structures includes the AFM and SEM measurements. The average roughness obtained from the AFM measurements presented in Table S2 and SEM images of the structures imaged in Figure S2. The R_a was calculated using program NT-MDT NOVA image analysis software.

materials

Table S2. The measured average roughness of the samples with and without rGO interlayer.

Sample	R_a 1 point (nm)	R_a 2 point (nm)	R_a 3 point (nm)	R_a 4 point (nm)	R_a 5 point (nm)	R_a 6 point (nm)	R_a 7 point (nm)	R_a 8 point (nm)	R_a 9 point (nm)	R_a 10 point (nm)	Average
1 QDs layer	8.2	8.2	6.6	6.6	6.8	8.5	6.3	7.1	8.2	7.9	7.4 ± 0.8
2 QDs layers	9.2	9.5	8.8	8.7	7.5	10.4	9.6	7.7	8.6	103	9 ± 1
rGO interlayer	10.2	10.8	12.4	10.8	12	9.1	10	8.6	10.2	10.4	10.5 ± 1.2
f-rGO interlayer	6.6	6.9	7.9	8.9	8.2	8.6	10.3	10.1	10.4	8.1	8.6 ± 1.3
f-rGO hybrid	13.1	10.7	21.3	11.1	10.6	8.7	18	6.7	9.3	7.4	11.7 ± 4.6

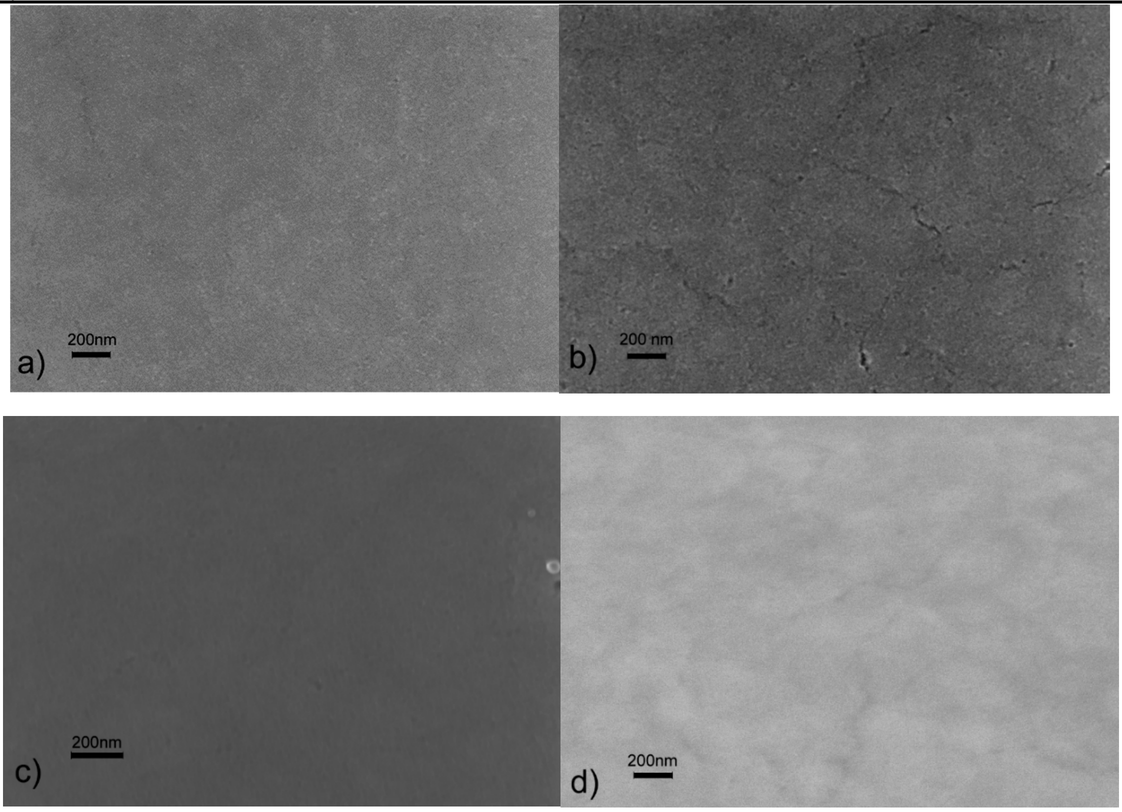

Figure S2. The SEM images of (**a**) TBAI treated QDs; (**b**) TBAI treated QDs with rGO interlayer; (**c**) TBAI treated QDs with f-rGO interlayer and (**d**) TBAI treated layers from f-rGO–PbS hybrid inks. The scale bar of all images is 200 nm.

The dark I-V curve of reference and device with rGO interlayers are imaged in Figure S3. The ideality factor was obtained by fitting curves in log I scale.

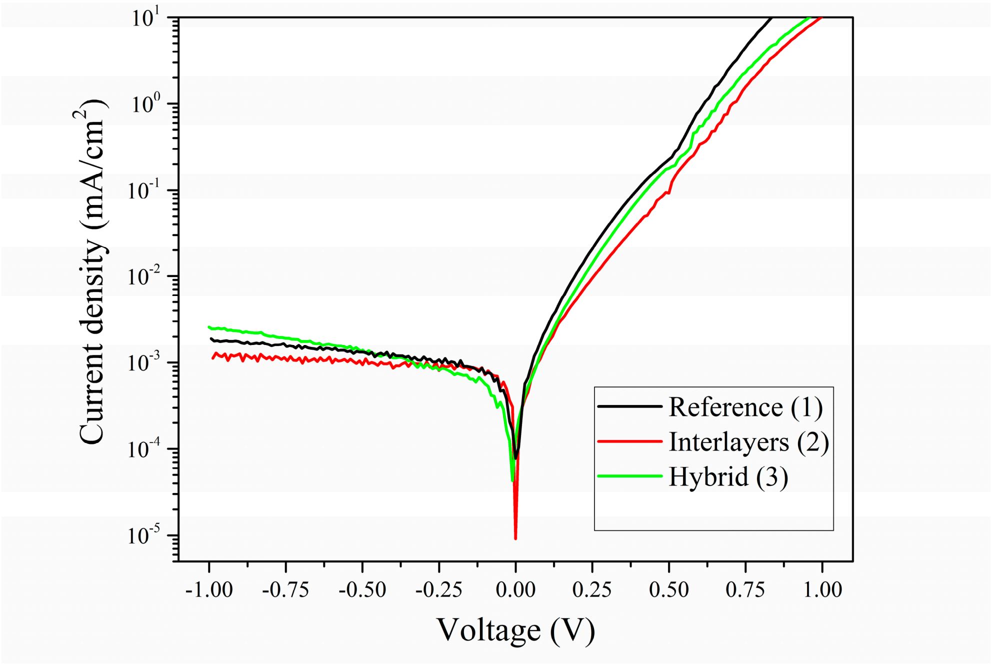

Figure S3. The dark I-V curves of the devices.

The dark current of the device is described by Equation (1) [3], that is, it exponentially depends on the voltage and constant ($\frac{e}{nkT}$), in which n is the ideality factor that characterizes the losses in the device. Logarithmizing Expression 1 we will turn into Expression 2 [4], where m = $\frac{\ln\frac{I}{I_0}}{V}$. The ideality factor can be easily extracted from Expression 2 by approximation of the I–V curve.

$$I = I_0\left[exp\left(\frac{eV}{nkT}\right) - 1\right], \quad (1)$$

$$m = \frac{1}{n}\frac{e}{kT}, \quad (2)$$

where I_0 is the saturation current; e is the elementary charge; V is the applied voltage; T is the temperature; n is the ideality factor, $m = \frac{\ln\frac{I}{I_0}}{V}$ is the slope of the curve, and k is the Boltzmann constant.

From theory for silicon solar cells, the value of n ranges from 1 to 2. The n = 1 corresponds to an ideal device with no losses and the larger values of n indicate the type of recombination in the device. However, for the 3rd generation solar cells the values of n can significantly exceed the silicon ones [5,6]. Factors that lead to the growth of n are the dependence of the barrier height on the applied voltage, a layer of natural oxide at the interfaces, electron tunneling through the barrier, high serial resistance of the neutral region, and the carriers recombination in the depletion zone of the device [7].

The C-F measurements are presented in Figure S4. The frequency of 10^4 Hz was chosen for correct C-V measurements.

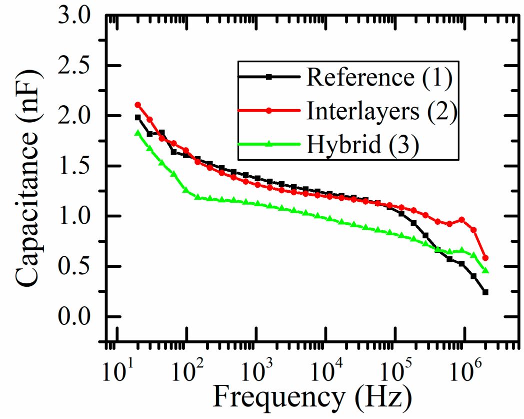

Figure S4. Capacitance of the devices at different frequencies.

Reference

1. Parfenov, P.S.; Litvin, A.P.; Ushakova, E. V.; Fedorov, A. V.; Baranov, A. V.; Berwick, K. Note: Near infrared spectral and transient measurements of PbS quantum dots luminescence. *Rev. Sci. Instrum.* **2013**, *84*, 116104.
2. Ushakova, E. V.; Litvin, A.P.; Parfenov, P.S.; Fedorov, A. V.; Artemyev, M.; Prudnikau, A. V.; Rukhlenko, I.D.; Baranov, A. V. Anomalous size-dependent decay of low-energy luminescence from PbS quantum dots in colloidal solution. *ACS Nano* **2012**, *6*, 8913–8921.
3. Esfahani, M.S. Device physics of organic and perovskite solar cells. Ph.D. Thesis, Iowa State University, Ames, IA, USA, 2015; ISBN 9789403404615.
4. Donald A. *Neamen Semiconductor physics and devices*; McGraw-Hill, New York, NY, USA, 2003; Volume 9; ISBN 0072321075.
5. Yoon, W.; Boercker, J.E.; Lumb, M.P.; Placencia, D.; Foos, E.E.; Tischler, J.G. Enhanced open-circuit voltage of PbS nanocrystal quantum dot solar cells. *Sci. Rep.* **2013**, *3*, 2225.
6. Gao, W.; Zhai, G.; Zhang, C.; Shao, Z.; Zheng, L.; Zhang, Y.; Yang, Y.; Li, X.; Liu, X.; Xu, B. Towards understanding the initial performance improvement of PbS quantum dot solar cells upon short-term air exposure. *RSC Adv.* **2018**, *8*, 15149–15157.
7. Çaldiran, Z.; Şinoforoǁlu, M.; Metin, Ö.; Aydoǁan, Ş.; Meral, K. Space charge limited current mechanism (SCLC) in the graphene oxide-Fe3O4 nanocomposites/n-Si heterojunctions. *J. Alloys Compd.* **2015**, *631*, 261–265.

© 2020 by the authors. Submitted for possible open access publication under the terms and conditions of the Creative Commons Attribution (CC BY) license (http://creativecommons.org/licenses/by/4.0/).
